# Drug-related HIV epidemic in Pakistan: a review of current situation and response and the way forward beyond 2015

**DOI:** 10.1186/s12954-015-0079-5

**Published:** 2015-10-16

**Authors:** Anne Bergenstrom, Baseer Achakzai, Sofia Furqan, Manzoor ul Haq, Rajwal Khan, Marc Saba

**Affiliations:** United Nations Office of Drugs and Crime (UNODC), Plot 5-11, Diplomatic Enclave-II, G-4, Islamabad, 44000 Pakistan; National AIDS Control Programme, National Institute of Health, Chak Shahzad, Park Road, Islamabad, 44000 Pakistan; United Nations Office of Drugs and Crime (UNODC), Plot 5-11, Diplomatic Enclave-II, G-4, Islamabad, 44000 Pakistan; UNAIDS Country Office for Pakistan & Afghanistan, Level-5, Serena Business Complex, Khyaban-e-Suhrwardy, Islamabad, Pakistan

**Keywords:** Injection drug use, Pakistan, HIV, AIDS, Hepatitis C, Prisoners

## Abstract

Pakistan is among four countries in Asia where the estimated number of new HIV infections has been increasing year by year ever since 1990. The Asian Epidemic Modelling (AEM), conducted in 2015, reconfirmed that the use of contaminated injection equipment among people who inject drugs (PWID) remains the main mode of HIV transmission in the country. The estimated number of PWID ranges from 104,804 to 420,000 PWID. HIV prevalence in this population is above 40 % in several cities, including Faisalabad (52.5 %), D.G. Khan (49.6 %), Gujrat (46.2 %), Karachi (42.2 %) and Sargodha (40.6 %), respectively. Harm reduction service delivery is being implemented through a public-private partnership led by the National and Provincial AIDS Control Programmes and Nai Zindagi with funding support from the Global Fund. Current programmatic coverage of the needle and syringe programme, HIV testing and counselling and antiretroviral treatment among PWID remain insufficient to control ongoing transmission of HIV in the country. While opioid substitution therapy (OST) is yet to be introduced, significant progress and coordination among various ministries have taken place recently to register buprenorphine in the dosage required for treatment of opioid dependence, and possible introduction of OST will greatly facilitate adherence to antiretroviral treatment among PWID living with HIV.

## Introduction

It has been nearly 30 years since the first AIDS case was diagnosed in Pakistan in 1986 [1]. Since then, transmission of HIV has continued in a slow but steady manner. Pakistan is among four countries in Asia, along with Indonesia, Malaysia and the Philippines, where the HIV epidemic is expanding with a number of new HIV infections higher each year than the previous year with an estimated 15,606 new infections in 2014 alone. Injection drug use remains a major mode of ongoing transmission of HIV in the country. According to the 2014 World Drug Report by United Nations Office of Drugs and Crime (UNODC), while it has been estimated that an average of 13.1 % of people who inject drugs (PWID) worldwide are living with HIV, HIV prevalence in this population is highest in South-West Asia (28.8 %), followed by Eastern and South-Eastern Europe (23.0 %), respectively, and the high prevalence of HIV among PWID in the South-West Asia region is predominantly accounted for by high prevalence of HIV among PWID in Pakistan. This paper reviews the epidemiology of and response to addressing drug-related HIV epidemics in Pakistan, drawing from published and unpublished literature.

## Review

### Epidemiology of injection drug use and HIV/AIDS among people who inject drugs and their intimate partners

An estimated 91,340 people (range 61,000–128,000) were living with HIV as of December 2014 and another 15,606 people (range 7000–33,000) became newly infected with HIV in that year [2]. While the overall prevalence of HIV among 15–49-year olds in the country has remained below 1 % (0.8 % in 2014), HIV prevalence among street-based PWID is among the highest reported in Asia and recent modes of transmission studies project an increase of transmission among men who have sex with men. A cohort study of 636 HIV seronegative PWID who were registered with three drop-in centres in Karachi found incidence of HIV to be 12.4 per 100 person-years (95 % exact Poisson confidence interval 10.3–14.9) which is high considering that the PWID were attending harm reduction services. Daily injection of drugs, sharing of syringes, being homeless and non-Muslim religion were found to be predictors of HIV seroconversion in the multivariable Cox Regression analyses [3].

While concerned stakeholders await for the findings of the fifth round of Integrated Biologic and Behavioral Surveillance Survey (IBBS) which is in the process of being implemented during 2015, the main source of information on behaviours that place PWID and their intimate partners at risk of HIV and other blood-borne viruses used to inform the national strategic planning and monitoring of the HIV response at the moment remains the IBBS which was conducted in 2011 [4].

In the 2011 round of IBBS, 4956 PWID were recruited and interviewed. Their average age was 30.4 years and 33.8 % were married while 57.1 % had no education. Majority (71.5 %) injected two to three times a day and most (90.5 %) had their last injection on streets or open spaces, whereas 80.9 % injected with friends and acquaintances. Only 38.6 % reported “always using a new syringe” for drug injection. Sharing of injection equipment for the last injection was reported by 39.2 % of the PWID. Sex with a regular female partner in the past 6 months was reported by 26.4 % of the PWID, and condom use at the last sexual act was reported by 25.8 %. Seven per cent PWID reported having had sex with a male sex worker or hijra sex workers in the past 6 months. Up-to-date information on HIV prevalence in this population is not available since the most recent sentinel survey was conducted in 2011 with the exception of programme data derived from HIV testing and counselling sites based on which the overall HIV prevalence among PWID was 33 % [5].

The 2011 IBBS also provides a glimpse of intermixing of key populations. Altogether, 10.1 % male and hijra sex workers had sex with PWID while 7.1 % PWID had paid a male or hijra sex worker for sex in the past 6 months. A sizeable proportion of FSWs (10.8 %) had sex with PWID in the previous 6 months. A total of 76 HIV samples were analysed from men who have sex with men (MSM), their female spouses and children, along with 26 samples from a previously studied cohort of PWID. Phylogenetic analysis of HIV gag gene sequences obtained from these samples indicated a substantial degree of intermixing between the PWID and MSM populations, suggesting a bridging of HIV infection from PWID, via MSM, to the spouses and children of MSM.

According to data from the 2011 IBBS, HIV prevalence among PWID has steadily increased from 10.8 % in 2005 to 27.2 % in 2011. HIV prevalence in this population is above 40 % in several cities, including Faisalabad (52.5 %), D.G. Khan (49.6 %), Gujrat (46.2 %), Karachi (42.2 %) and Sargodha (40.6 %), respectively. The latest round of IBBS in the province of Punjab, conducted in 2014, indicated a weighted prevalence of 36.8 % among ten cities of Punjab. Based on a model by Reza et al. [6], HIV prevalence may increase to 65–75 % among PWID in Faisalabad, 46–66 % among PWID in Karachi, 44–49 % among PWID in Lahore by 2015 and the total number of people who inject drugs infected with HIV may reach 68,000 by 2020.

Intimate partners of men who inject drugs are especially vulnerable to acquiring HIV infection. For example, within a short space of time after the initial HIV epidemic outbreak among men who injected drugs in Manipur, Northeast India, in 1989, HIV prevalence among wives of the men had reached 45 % [7]. In Pakistan, information about HIV risk and vulnerability of female spouses of PWID is available from a cross-sectional survey of men, and their spouses, who were accessing street-based harm reduction services provided by Nai Zindagi in three cities of the country. The survey of 459 husband and wife couples revealed 28 % HIV prevalence among the male PWID and 15 % HIV prevalence among their wives in Faisalabad, 10 % in Lahore and 5 % in Sargodha, respectively. Since none of the women interviewed reported having injected drugs, the main mode of HIV transmission among the women was assumed to be sexual transmission from their husbands. On average, couples that were surveyed had four children, 20 % were breastfeeding and 8 % were pregnant at the time of the survey illustrating the high risk of non-injecting spouses and their babies in acquiring HIV [8]. The most recent data on HIV prevalence among wives of PWID is available from programme data collected from the 28 sites which deliver harm reduction services with funding support from the Global Fund to Fight AIDS, Tuberculosis and Malaria (GFATM). Among female spouses of PWID, 7 % of the spouses were found to be infected with HIV with the highest prevalence in Balochistan (29 %) [9].

### Prevalence of HBV and HCV among PWID

Data on prevalence of hepatitis B and hepatitis C among PWID in Pakistan is scarce. The latest available data is from 2003 to 2004 at which time 6.8 % (range 6.0–7.5 %) of PWID were found HBsAg positive and 84.0 % (range 75.0–92.9 %) HCV positive, respectively [10]. A cross-sectional study of 161 PWID conducted in 2003 in Karachi found 94.3 % hepatitis C positive while prevalence of hepatitis B was 7.5 % [11].

Discussions are underway regarding the need to assess current prevalence of HCV among PWID. In a descriptive observational study of 162 HIV-positive men, including 81 PWID and 81 men who did not inject drugs, conducted at an HIV treatment and care centre at the Pakistan Institute of Medical Sciences (PIMS) hospital, Islamabad, 66 (77.8 %) of PWID tested positive for HBsAg, while only five (6.2 %) of the patients who did not inject drugs were found to have hepatitis C [12].

### HIV among prisoners

As of December 2013, there were 104 prisons in Pakistan with a total capacity for 45,587 prisoners. The actual number of prisoners as of 31 December 2013 was 77,504 (170 % of the authorised accommodation) among whom 2408 (3.1 %) were “drug addicts/users” [13]. Drug use, including injection drug use, and unprotected sexual behaviour resulting in transmission of HIV and other blood-borne infections in prisons cannot be ruled out presenting significant challenges for prison management and public health authorities.

For example, following screening of 43,000 prison inmates in Punjab province in 2009, 231 (0.54 %) were found infected with HIV [14]. In another study in one prison in Punjab, 4.4 % of 1000 prisoners tested HIV positive while prevalence of HCV was 19.7 %, with 3.7 % co-infected with HIV and HCV [14].

In 2012, UNODC Pakistan Country Office commissioned a bio-behavioural study in five prisons in Sindh province with the objectives to explore HIV risk and vulnerability among prisoners as well as to assess HIV prevalence among the prison inmates [15]. A total of 1198 interviews were conducted with inmates all of whom were tested for HIV. Opium and heroin use “ever” was reported by 15 and 16 % of the inmates, respectively, while of those reporting drug use, 32.9 and 50.9 % reported having used drugs in prison. “Ever use” of injection drugs was reported by 9.8 % of the inmates. Injection drug use within prisons was reported by 12.3 % among those who reported having ever injected drugs. The overall HIV prevalence across the five prisons was 2.3 %, considerably higher than the overall national prevalence of HIV among adults (15–29) which is <0.1 %. Central Jail Hyderabad reported the highest HIV prevalence among the prisoners at 3.7 %, followed by 2.9 % in Central Jail Sukkur.

### Extent and nature of drug use

In 2013, a national survey on drug use was conducted by the Pakistan Bureau of Statistics, in coordination with the Narcotics Control Division and with support from UNODC and the United Nations Country Team [16]. The national survey consisted of five components: a household survey of 51,000 respondents randomly selected in households across the country; a problem drug use survey, consisting of interviews with 4500 individuals who had used illicit substances on a regular basis (in the past 12 months and 30 days) for non-therapeutic purposes in 23 districts; interviews with 1200 key informants (23 districts); an audit of drug treatment services (across the country); and an audit of drug-related arrest data. The interviews with the “problem drug users” were conducted in community settings using snowball sampling strategy.

The findings from the household survey indicated that an estimated 860,000 people, or 0.8 % of the population aged 15–64, used heroin regularly. Based on the findings from the problem drug use survey, an estimated 430,000 (range 190,000 to 657,000), or 0.4 % (range 0.3 to 0.5 %) people aged 15–64, inject drugs. Among these, 78 % injected heroin and the remaining injected pharmaceutical opioids, including morphine, pentazocine or pethidine, and tranquilisers for non-therapeutic purposes. Poly drug use was a common phenomenon with over 75 % of opioid users injecting a combination of substances. In this survey, the majority reported injecting two to four times a day.

According to the 2015 Spectrum, the number of PWID was estimated at 104,804. This estimate is based on an extrapolation of data from the mapping that was conducted as part of the 2011 IBBS. For the purpose of consistency of methodology over time, the National AIDS Control Programme is using the 2015 Spectrum PWID size estimate to inform relevant national planning processes, such as the development of Pakistan AIDS Strategy III and the HIV Concept Note which was submitted to the Global Fund in April 2015.

## Status of the current harm reduction response

### Policy, strategy and legal framework

In September 2007, the HIV & AIDS Prevention, and Treatment Act, was finalised. The Act aims at “preventing the HIV from becoming established in the general population, particularly in the most-at-risk and vulnerable populations, and to provide for the care, treatment and support of persons living with HIV and AIDS”.

The Government of Pakistan developed a National HIV and AIDS Policy in 2007 [17] which remains in draft form. The HIV and AIDS Policy notes that the focus of HIV prevention efforts needs to be on “groups most affected by HIV and AIDS: injecting drugs users and people who engage in sexual behaviour that puts them at risk”. The prevention strategies, envisaged in the Policy include “needle and syringe outreach to injecting drug users and referral to drug treatment services”. The National HIV and AIDS Strategic Framework 2007–2012 aimed at “translating the National Policy on HIV and AIDS by providing strategic guidance to the planning of programmes, projects and interventions by various stakeholders” [18]. Following the 18th Amendment and the subsequent devolution in 2011, the Ministry of National Health Services, Regulation and Coordination was devolved to the provinces. Since devolution, the Provincial AIDS Control Programmes developed Provincial AIDS Control Strategies which are available in all four provinces. One of the three outcome areas in each of these strategies relates to reduction of HIV Prevalence among Key Affected Populations (including prisoners) maintaining prevalence at below 0.1 % in the general population.

The Ministry of Narcotics Control of the Government of Pakistan has a Drug Control Master Plan (2010–2014) and a National Anti Narcotics Policy (2010) updated in August 2011. The objectives of the Drug Control Master Plan and National Anti Narcotics Policy are threefold: (i) supply reduction, (ii) demand reduction and (iii) international cooperation. With regard to demand reduction, a key focus of the National Anti Narcotics Policy is “enhancing demand prevention efforts focusing through education and community mobilisation campaign and projects”, including to “develop special training and treatment programmes on drug abuse and drug related HIV/AIDS prevention/rehabilitation” in jails. Furthermore, the National Anti Narcotics Policy notes that an estimated 40 % of Pakistan’s prison population use drugs. In view of the scale of drug use among prisoners, the Policy notes that, in addition to drug demand reduction efforts, “drug treatment and rehabilitation facilities will be extended to key jails”.

### Availability and programme coverage of recommended HIV services among PWID

Under the leadership of the National AIDS Control Programme (NACP), the Provincial AIDS Control Programmes (PACPs), in partnership with non-governmental organisations, are implementing several of the nine recommended HIV prevention, treatment and care services at community level [19], including needle and syringe programme, HIV testing and counselling and referral to antiretroviral treatment, care and support for those who test HIV positive. The national HIV response is implemented in close cooperation between the NACP, PACPs, UN agencies, NGOs and CSOs with the support of donor partners.

Needle and syringe programme (NSP) has been operational since 2003–2004 (NSF II) in Pakistan. The programme is implemented by the Provincial AIDS Control Programmes and Nai Zindagi, one of the two principal recipients (PRs) of the Global Fund grant for harm reduction from the round 9 as well as a range of a sub-recipient NGO implementing partners. In Punjab province, the Provincial AIDS Control Programme is implementing NSP in 9 cities. With support from the round 9 funding, Nai Zindagi is covering another 13 cities/districts in Punjab province, 10 cities/districts in Sindh province and 2 cities in Balochistan province, respectively. Data from a total of 28 sites supported by the GFATM indicate that there were some 39,935 PWID, or some 38 % of the estimated 104,804 PWID in the country, registered with harm reduction programmes supported by the GFATM between 2012 and 2014 [20]. A total of 1484 spouses of PWID were also registered and receiving services.

As per the programme monitoring data, the number of needles and syringes per PWID per year has increased from 131 per PWID per year in 2011 to 470 per PWID per year in 2014 [21]. Several factors, including drug use-related arrests by police, continue to hamper expansion of the needle and syringe programme. For example, in a survey of HIV treatment access among 545 people who use drugs by the Association of People Living with HIV/AIDS (APLHIV) in 2013 and 2014, 48.3 % reported having been arrested or detained for drug use [22].

Comparison of behavioural and HIV prevalence data among PWID from the 2011 IBBS in cities that have harm reduction interventions and in cities without intervention found higher rates of use of sterile needles and syringes (59 vs. 27 %), higher rates of condom use (24 vs. 11 %) and higher levels of HIV knowledge in the intervention cities [23]. However, HIV prevalence among PWID in the intervention cities remained unchanged in the city of Faisalabad (13 %), and prevalence was found to have increased in Karachi (26–30 %) and Lahore (4–7 %) [23]. Low level of programmatic coverage of harm reduction services is considered among the main factors in fuelling the HIV epidemic among street-based injectors.

People who inject drugs are disproportionally less likely to receive treatment for their HIV infection compared with people who do not inject drugs, as reported in a global review of coverage of antiretroviral therapy (ART) among PWID [24]. One of the underlying reasons for the low uptake of and access to ART in this population is low uptake of HIV testing among PWID which in Pakistan remains very low compared with other countries in Asia. The 2011 IBBS indicated that 9.1 % of PWID had tested for HIV in the previous 12 months from having been interviewed. Of the 11,038 registered people living with HIV, 5019 (45.5 %) were receiving antiretroviral treatment as of the end of December 2014. While the 2015 Spectrum estimates that 54,541 adults and 1835 children would be eligible for ART, the ART coverage among adults in need of treatment is 9.2 % and children in need of treatment is 5.6 %, respectively.

In a survey of 545 people who use drugs by the Association of People Living with HIV (APLHIV), 298 (54.7 %) had tested for HIV while the number of study respondents who reported being on ART was only 23 (4.2 %) despite a high overall prevalence of HIV in the population of people who use drugs [22]. In the same survey, 25.3 % (*n* = 138) reported having been denied access to medical care “in the recent past” and 28.1 % (*n* = 153) had been denied admission in hospital.

Once on ART, adherence to HIV medicines among PWID remains a challenge and is increasingly raising concerns about possible resistance to antiretroviral (ARV) drugs , including second line ARVs. It is well documented that people who use drugs are especially vulnerable to poor adherence to antiretroviral therapy [25] which is not only a barrier to viral suppression in the HIV-positive individual but also negatively impacts on prevention of onward transmission of HIV at population level [26]. Available data on adherence to ART among PWID in Pakistan also indicates lower adherence to ART among PWID compared to HIV patients who do not inject drugs. A prospective study of 162 HIV-positive male patients, including 81 PWID and 81 patients who did not inject drugs, in an ART centre in a major public hospital in Islamabad over a 5-year period found that 41 (50.6 %) of the PWID were lost to follow-up compared with two (2.5 %) of the non-injection drug users who were lost to follow-up at the end of the study. Furthermore, the HIV treatment adherence levels ranged from 90.1 % among patients who did not inject drugs compared to 19.8 % among HIV patients who injected drugs, respectively [27]. Another recent survey in Jinnah Hospital in Lahore found that, of the 869 patients who had ever been registered with the ART clinic, 273 (31.4 %) were PWID. Only 32.6 % of the HIV-positive PWID had ever been put on HIV treatment compared with 54 % of the non-PWID. Over a 6-month follow-up period, 25 % of the PWID who were registered with the ART clinic continued on treatment while the rest were lost to follow-up [27].

A meta-analysis of 38 studies that assessed adherence to ART among people who use drugs living with HIV found higher adherence among patients who were receiving opioid substitution therapy (OST) [28]. OST, a key component of the comprehensive package of nine HIV services recommended by WHO, UNODC and UNAIDS, is yet to be introduced and scaled up in Pakistan. As of now, Pakistan is the only country in Asia with large-scale use of opioids that does not have a national OST programme. Yet, leading HIV specialist physicians in the country report that absence of OST, which would help to stabilise HIV patients majority of whom are people who inject drugs, is one of the main barriers to uptake of and adherence to HIV treatment among HIV-positive PWID. While OST was reflected and funding earmarked in the round 9 of the GFATM proposal; it was not possible for the principal recipients to implement the activity due to the absence of an enabling policy environment for the intervention.

A Technical Steering Committee (TSC) for opiate substitution therapy (OST) was established by the Ministry of National Health Services (M/o NHSR & C), Regulations and Coordination in 2013. The Chairman of the TSC was Secretary, M/o NHSR & C and Vice Chair was Director General, M/o NHSR & C. The National AIDS Control Programme was the designated Secretariat of the TSC. Members of the TSC included representatives of the Narcotics Control Division, Anti Narcotics Force, Drug Regulatory Authority, Managers of the Provincial AIDS Control Programmes, eminent psychiatrists, Nai Zindagi, representative of the drug user community, UNAIDS, UNODC and WHO.

Under the auspices of the OST TSC, the WHO-UNODC Joint Programme on Drug Dependence Treatment and Care supported a pilot study on the use of buprenorphine for treatment of opioid-dependent people in 2013. The pilot feasibility study was implemented by the Institute of Psychiatry and the Narcotics Control Division in partnership with the National HIV/AIDS Control Programme, UNODC Country Office Pakistan, WHO and UNAIDS. A total of 210 individuals enrolled in the pilot of whom 80 were availing of the OST programme as of December 2013. Analysis of the baseline and follow-up data found a significant reduction in heroin use and injecting among patients receiving treatment as well as significant improvements in health and quality of life among those on treatment. One of the major challenges faced by the pilot project was unavailability of buprenorphine in the required strength as absorption of a large number of crushed tables sublingually was a practical challenge for the patients. Despite the challenges, 73 % of the patients were retained in treatment at 12 months.

Despite ongoing advocacy on the importance of introduction and expansion of OST for the purpose of reducing demand for injecting of opiates, treatment of dependence on opioids and for control of HIV and hepatitis C transmission, pharmacologically assisted treatment of dependence on opioids remains unavailable as of now. Possible introduction of pharmacologically assisted treatment will require approval of registration of buprenorphine in the required dose of 2 and 8 mg sublingual tablets by the Drug Regulatory Authority of Pakistan (DRAP) which is currently under consideration by the DRAP.

Following a mid-term review of the Provincial AIDS Control Strategies, the National AIDS Control Programme has finalised the Pakistan AIDS Strategy III (PAS III). The PAS III recognises the important role of OST in HIV prevention and treatment. As part of the PAS III, a separate strategy for OST has been developed by the NACP, with support from UNODC, UNAIDS and WHO. The OST strategy, which has been reflected in the PAS III and the HIV Concept Note for the Global Fund, proposes a phased approach to introduction and expansion of OST consisting of (i) phase 1—preparatory phase (2015 through to mid 2016), (ii) phase 2—implementation of OST service delivery (mid 2016–December 2017) and (iii) phase 3—scale up and implementation of OST service delivery in an additional 13 cities (2018–2020).

### Availability of HIV services in prisons

Access to HIV prevention, treatment and care services, recommended by UNODC, ILO, UNDP, WHO and UNAIDS [29], is limited and mostly focused on general awareness raising among prisoners and prison management staff. A scan of service provision in prisons in 2014 by UNODC found that ART was available on a limited scale for prisoners in some prisons in two out of four main provinces. For example, in Punjab province, 42 prisoners were receiving ART, and in Sindh province, six prisoners were on ART as of April 2014. Condoms were reportedly available in prisons in Sindh province while needle and syringe programme remains unavailable in prisons. A series of capacity building training workshops have been organised by UNODC during 2013 and 2014 with a total of 150 prison management staff, including prison administration staff and psychologists having been trained on prevention of drug use and HIV prevention in prisons in the four main provinces. Following organisation of a national coordination meeting on HIV services in prisons in 2014, UNODC Pakistan Country Office, in coordination with the NACP, will be supporting organisation of provincial stakeholder consultations on prisons and HIV, aimed at discussing and agreeing on expansion of HIV services in prisons building on what is already being implemented in prisons.

## Conclusions

A key component of the comprehensive package of services, opioid substitution therapy (OST), recommended by WHO, UNODC and UNAIDS, is yet to be introduced and scaled up in Pakistan. Given that an estimated 430,000 people inject drugs, mostly opiates, introduction and expansion of OST that is accessible to large numbers of people who have developed dependence on opioids is critical. The absence of OST, which would help to stabilise ART patients and promote adherence to ART among patients majority of whom are PWID, is one of the main barriers to treatment adherence.

Ensuring access to and adherence to ARVs among people living with HIV, especially those who inject drugs, has the potential to prevent much of the ongoing transmission of HIV in Pakistan. Given low rates of HIV testing and counselling overall, and among PWID in particular, there is a need to explore new approaches that will attract much larger numbers of PWID to test for HIV so that those who test positive can benefit from treatment. Eventual introduction of OST will greatly improve adherence to ART, leading to the suppression of viral load among HIV-positive PWID and consequently averting new cases of transmission of HIV from this population.

The voice of people who inject drugs is mostly silent in national policy making, planning, implementation, monitoring and evaluation processes. With support from the International Network of People who use Drugs (INPUD) and Mainline, The Association of People Living with HIV/AIDS (APLHIV) has in 2015 launched a Drug Users Network (“DUNE”). The mission of DUNE is to mobilise community leadership in order to influence laws, policies, programmes and funds through meaningful involvement of people who use drugs. It is anticipated that the formation of such a national network will enhance the representation and voice of people who use drugs in relevant fora.

The PAS III has prioritised prevention, treatment and care services for key populations, including PWID and prisoners. This approach remains the cornerstone of Pakistan’s HIV response proposed for funding by the Global Fund in addition to the financial resources that are available through the provincial and national “PC1s” (costed work plans). While the epidemic remains concentrated among key populations and their networks, there is no room for complacency given the large population of PWID and continued high prevalence of HIV among PWID in the country.
